# Transcriptome profiling of neurexin- and neuroligin-deficient Caenorhabditis elegans reveals pathways underlying developmental and behavioral dysfunction

**DOI:** 10.21203/rs.3.rs-10280322/v1

**Published:** 2026-07-08

**Authors:** Omamuyovwi Ijomone, Victor Anadu, Toheeb Oyerinde, Olayemi Ijomone, David Oyeniran, Michael Aschner

**Affiliations:** Albert Einstein College of Medicine; Albert Einstein College of Medicine

**Keywords:** Caenorhabditis elegans, Synaptic dysfunction, Neurexin and Neuroligin, Transcriptomics, Neurodevelopmental disorders

## Abstract

Neurexins and neuroligins are evolutionarily conserved synaptic adhesion molecules that play essential roles in synapse formation and neural circuit function, with mutations linked to neurodevelopmental disorders such as autism. Here, we combined whole-transcriptome sequencing with phenotypic characterization to define the molecular consequences of neurexin and neuroligin deficiency in *Caenorhabditis elegans*. Young adult worms carrying allele-specific loss-of-function mutations in *nrx-1* (*ok1649* and *tm1961*) or *nlg-1* (*ok259* and *tm474*), orthologues for human *NRXNs* or *NLGNs*, respectively, were subjected to RNA sequencing and compared with wild-type animals. Mutant strains exhibited impaired growth, altered locomotor activity, increased social aggregation, and reduced ventral nerve cord neuronal integrity. Transcriptomic analysis revealed extensive gene-expression changes, particularly in the *nrx-1* (*tm1961*) allele, with dysregulation of genes involved in cuticle development, neuronal signaling, protein homeostasis, innate immunity, mitochondrial organization, and transcriptional regulation. Gene Ontology and KEGG enrichment analyses identified significant perturbations in developmental, metabolic, stress-response, translational, and synaptic pathways. Together, these findings demonstrate that disruption of neurexin–neuroligin signaling drives transcriptional reprogramming that extends beyond synaptic dysfunction, linking molecular alterations to developmental, behavioral, and neuromorphological abnormalities.

## Introduction

The brain is one of the most complex biological systems, responsible for coordinating cognition, behavior, and adaptive responses to the environment. Its functional complexity arises not only from the total number of neurons it contains, but more critically from the intricate synaptic connectivity between them^1,2^. This connectivity supports efficient information processing, neural plasticity, and the emergence of complex neural networks^3,4^.

Central to the organization of structural and functional integrity of synapses are cell adhesion molecules (CAMs), a diverse class of transmembrane glycoproteins that mediate trans-synaptic interactions and coordinate synapse formation, specification, and plasticity^5^. These molecules not only physically connect pre- and postsynaptic membranes but also initiate intracellular signaling cascades that organize synaptic machinery^6^. Among these CAMs, neurexins and neuroligins are the most extensively studied and functionally characterized pair, forming a canonical trans-synaptic complex essential for synaptic development and function^7^.

Neurexins are predominantly presynaptic adhesion molecules encoded by three genes, *NRXN1*, *NRXN2*, and *NRXN3*, which generate α- and β-isoforms through alternative promoters and extensive alternative splicing^8,9^. This structural diversity enables neurexins to participate in a wide range of synaptic interactions, contributing to synaptic specificity^10,11^. Neuroligins, in contrast, are postsynaptic adhesion proteins encoded by *NLGN1*, *NLGN2*, *NLGN3*, and *NLGN4* (X/Y). They are localized to the postsynaptic membrane, where they interact with intracellular scaffolding proteins such as PSD-95 via PDZ-binding motifs, thereby organizing receptor complexes and postsynaptic density architecture^12,13^. Neuroligins also form Ca^2+^-dependent trans-synaptic complexes with neurexins through laminin G domains, a process that is essential for synapse assembly, maturation, and plasticity^14–16^. Together, these molecules ensure both structural stability and functional efficiency at synapses and play a crucial role in maintaining the balance between excitatory and inhibitory signaling. Disruption of this balance is a hallmark of many neurodevelopmental disorders^17,18^.

Alterations in neurexin and neuroligin function have been strongly implicated in neurodevelopmental disorders, particularly autism spectrum disorder (ASD, autism), which is increasingly viewed as a synaptopathy characterized by disrupted synaptic structure and function^19,20^. Genetic studies have identified mutations, deletions, and copy number variations in *NRXN* and *NLGN* genes in individuals with autism, intellectual disability, and related conditions^21^. Among these, deletions in *NRXN1* represent one of the most consistently replicated genetic risk factors and are associated with deficits in synaptic transmission and neural connectivity^22^. Similarly, mutations in *NLGN3* and *NLGN4* were among the first to be directly linked to autism, with the R451C substitution in *NLGN3* serving as a well-characterized example that alters synaptic function and behavior^23,24^.

Insights from animal models have been instrumental in elucidating the neurobehavioral consequences of these mutations. In mice, deletion of neuroligin genes has been shown to result in significant impairments in synaptic transmission and associated behaviors, despite relatively normal synapse numbers^25,26^. Knock-in models carrying autism-associated mutations, such as the *NLGN3* R451C variant, exhibit altered inhibitory synaptic transmission and behavioral abnormalities, including deficits in social interaction and changes in learning and memory^27–29^. Transcriptomic analyses of autism brains reveal dysregulation of synaptic adhesion pathways, including those involving neurexins and neuroligins, linking these alterations to changes in synaptic function, plasticity, and behavior^30^. The variability in clinical phenotypes associated with these mutations reflects the complexity of synaptic organization, where circuit-specific disruptions can lead to diverse neurodevelopmental outcomes^31–33^. Additionally, studies have shown that mutations leading to increased *NLGN4* expression can be detrimental, with overexpression associated with a spectrum of social and cognitive abnormalities ranging from Asperger syndrome to severe intellectual deficits and tics^21,34^

Despite substantial progress in understanding the roles of neurexins and neuroligins in synaptic formation and maintenance, a clear understanding of how they drive perturbations that trigger pathogenesis and progression of developing brain diseases remains elusive. In this context, it becomes necessary to leverage simple *in vivo* model systems, such as the innovative *Caenorhabditis elegans*, to tease apart the complex mechanistic basis. Comparative studies across model organisms further demonstrate that the functions of neurexins and neuroligins are evolutionarily conserved. The *C. elegans* has been shown to possess single orthologs of these genes (*nrx-1* and *nlg-1*), and their mutations have been shown to reduce synapse number, impair receptor localization, and disrupt synaptic plasticity and behavior, particularly sensory and social responses^35,36^. In this study, we explored transcriptomic changes in C. elegans carrying loss-of-function mutations in neurexin (nrx-1; alleles ok1649 and tm1961) and neuroligin (nlg-1; alleles ok259 and tm474), and supported these findings by characterizing developmental, behavioral, and neuromorphological phenotypes. These ASD-relevant mutant strains were systematically compared with wild-type animals to elucidate gene-specific effects.

## Results

### Nrx-1 and nlg-1-deficient worms show impaired growth and locomotion, and exaggerated repetitive movement

To evaluate the worm's physical growth (developmental) rate, we quantified the body length of worms deficient in *nrx-1 (ok1649), nrx-1 (tm1961)*, *nlg-1 (ok259)*, and *nlg-1(tm474)* compared to N2 (WT) at the young adult stage.

**Figure 1:**
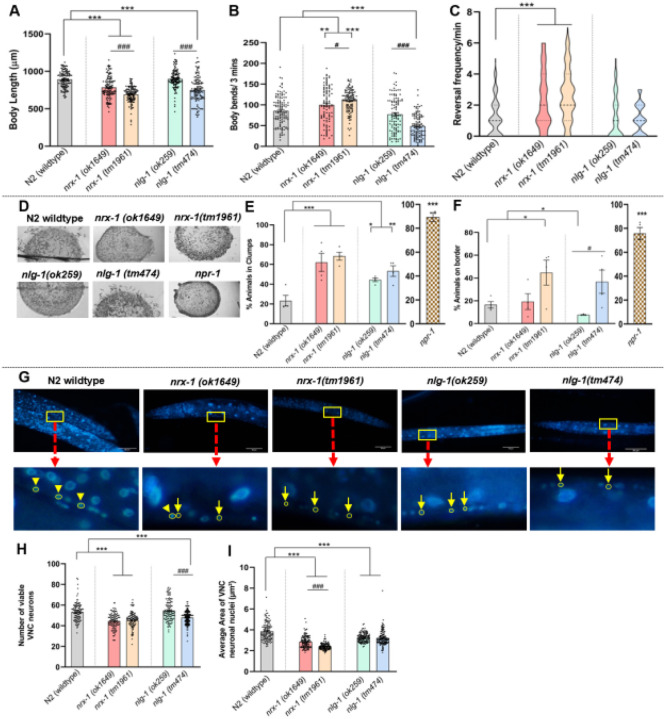
Growth rate, behavioral (motor, repetitive, and social), and neuromorphological phenotypes in *nrx-1* and *nlg-1* mutant *C. elegans*. **(A)** Reduced growth rate assessed by body length in young adults was observed in both *nrx-1* alleles compared to N2 (wild-type, WT), but only in the *nlg-1(tm474)* allele. Significant inter-allelic differences were detected within both *nrx-1* and *nlg-1* mutants. **(B)** Opposing effects are seen in locomotor activity, with *nrx-1* mutants showing increased activity (hyperlocomotion), whereas *nlg-1* mutants displayed reduced locomotion. **(C)** Both *nrx-1* alleles, but not *nlg-1* mutants, exhibited increased repetitive behavior. **(D–F)** Representative micrographs of worm social (bordering and clumping) behaviors; *npr-1* loss induces social aggregation and is shown for comparison with N2 **(D)**. Mutant strains exhibited a significant increase in clumping **(E)**, as well as increased bordering, which was more pronounced in *nrx-1 (tm1961)* and *nlg-1 (tm474)* **(F)**. **(G-I)** Representative fluorescence micrographs of DAPI-stained worms. Boxed regions show magnified views of ventral nerve cord (VNC) neurons. Arrowheads indicate viable neurons, while arrows indicate abnormal neurons **(G)**. Mutant strains exhibited a significant reduction in both the number **(H)** and size (area; **I**) of viable VNC neurons. *P<0.05, **P<0.01, ***P<0.001 compared to WT; ^#^P<0.05, ^##^P<0.01, ^###^P<0.001 comparing two alleles of the same strain (One-way ANOVA with Dunnett’s post hoc, and t-test where applicable).

We observed a significant decrease in body length in both *nrx-1* mutant strains relative to N2 (WT), with *nrx-1* (*tm1961*) exhibiting a more pronounced effect (Fig. 1A). On the other hand, only the *nlg-1* (tm474) mutant displayed a significant decrease in body length compared to N2. To test the general locomotion of worms, we scored the number of body bends off food over a 3-min interval. Hyperlocomotion was observed in *nrx-1 (ok1649)* and *nrx-1 (tm1961)* mutant strains when compared to N2. Conversely, the *nlg-1* mutant strains exhibited a significant reduction in body bends. Finally, we measured repetitive locomotion by scoring “head-tail-head” movements. We found that *nrx-1* mutant strains showed a significant increase in repetitive actions. However, there are no changes observed in *nlg-1* mutant worms when compared to N2 (WT).

#### C. elegans social behavior is altered in nrx-1 and nlg-1 loss-of-function mutants:

Clumping and bordering are sensitive social behavioral parameters in *C. elegans*. Naturally, *C. elegans* feed solitarily and disperse across the bacterial lawn; however, under certain genetic or environmental stressors, they exhibit altered behaviors: 'clumping,' where animals aggregate in clusters on the lawn, and 'bordering,' where they accumulate at the edge of the lawn (Fig. 1D). To investigate how alterations in *nlg-1* and *nrx-1* influence these social phenotypes, we quantified the clumping and bordering behaviors of mutants compared to N2 (WT) (Fig. 1E–F). Our results demonstrated a significant increase in clumping activity across all mutant strains compared to the N2 control. Regarding bordering behavior, both *nrx-1* mutant strains and the *nlg-1 (tm474)* mutant exhibited a significant increase in bordering frequency (*P < 0.05*). Conversely, *nlg-1 (ok259)* displayed a distinct phenotype, showing a significant reduction in bordering activity compared to the wild type.

### Loss of *nrx-1* and *nlg-1* function alters morphological features of VNC neurons

To support our behavioral findings, we evaluated neuronal integrity using DAPI staining. This fluorescent dye binds strongly to DNA and is commonly used to visualize and quantify cells, as well as assess nuclear integrity. The organization and spatial distribution of neuronal nuclei within the ventral nerve cord (VNC) are essential for preserving neuronal connectivity and functional integrity. Neurons and their nuclei within the VNC of *C. elegans* offer simple, yet important insights into the molecular and cellular mechanisms that govern neuronal development, function, and plasticity^37^. Our results demonstrate that loss of *nrx-1* and *nlg-1* functions reduced the number of viable VNC neurons (Fig. 1H, P *< 0.001*). Furthermore, morphometric analysis revealed a significant reduction in the average VNC neuronal nuclear size (area) in both *nrx-1* and *nlg-1* mutant worms (Fig. 1G, 1I, P *< 0.001*). These findings suggest that the loss of these synaptic genes predisposes worms to an altered ventral nerve cord morphology.

### Distribution of read counts in RNA-Seq analysis

Read counts were examined before and after normalization. The original counts were normalized to adjust for factors, including variations in sequencing yield between samples. These normalized read counts were used accurately to determine differentially expressed genes. Supplementary Fig. 1 (S1) shows the read counts for each sample before and after the normalization.

### Impact of *nrx-1/nlg-1* loss-of-function (LOF) on gene expression profile in *C. elegans*

**Figure 2:**
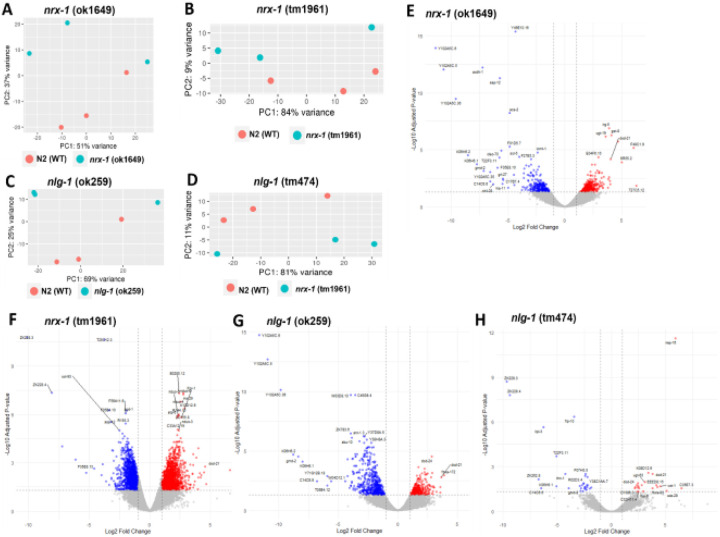
Differentially expressed genes (DEGs) in all mutant strains compared to N2 (WT). **(A-D)** PCA plots of all mutant strains showing similarity between respective samples based on the distance matrix. **(E-H)** Volcano plot of all DEGs. Red dots represent upregulated DEGs, blue dots represent downregulated DEGs, while grey dots indicate genes with no significant expression changes.

Gene expression profiles of worms with LOF mutation in *nrx-1 or nlg-1* follow total RNA-Seq revealed that, compared to the N2 wildtype control, *nrx*-1 (ok1649), *nrx*-1 (tm1961), *nlg*-1 (ok259), and *nlg*-1 (tm474) exhibited 448, 1912, 352, and 21 significantly upregulated genes, respectively, and 261, 1497, 506, and 30 significantly downregulated genes, respectively. Among these differentially expressed genes (DEGs), a total of 4 were consistently upregulated and downregulated across all mutant strains compared to the wild-type control, resulting in 8 genes showing consistent expression changes (Fig. 2E–H; Supplementary Figs. 2–3 (S2, S3)).

### *Nrx-1/nlg-1* loss downregulates genes encoding developmental and structural organization processes

The mutant strains showed downregulation of genes in the unfolded protein response (UPR), which encodes proteins active at the endoplasmic reticulum membrane, respond to stress, and are involved in pharynx development. Notably, *abu-1*, *abu-4*, *abu-6*, *abu-7*, *abu-8*, *abu-10*, *abu-11* genes were downregulated in *nrx-1* (ok1649) strain, *abu-6*, *abu-7*, *abu-8* in *nrx-1* (tm1961), and *abu-6*, *abu-7*, *abu-8*, *abu-10*, *abu-11*, *abu-15* in *nlg-1* (ok259). Furthermore, collagen genes which are parts of collagen trimer and are involved in collagen and cuticulin based cuticle development were consistently downregulated in the *nrx-1* (ok1649) [*col-157, col-170*], *nrx-1* (tm1961) [*col-76, col-92, col-93, col-94, col-124, col-139, col-155, col-159, col-39*], and *nlg-1* (ok259) [*col-10, col-34, col-39, col-92, col-109, col-118, col-125, col-130, col-133, col-144, col-145, col-147, col-155, col-157, col-168, col-169, col-170, col-186*] strains. The *nrx-1*(tm1961) strain also had downregulation of actin genes (*act-2, act-3, act-4, act-5*), which are involved in cytoskeleton organization and are predicted to be structural constituents of the cytoskeleton.

#### Nrx-1/nlg-1 mutations alter genes regulating stress response and homeostasis:

Notably, upregulation in genes involved in stress responses was observed across strains, including glutathione S-transferase in *nrx-1* (ok1649) [*gst-5, gst-6, gst-8, gst-12, gst-13*], *nrx-1* (tm1961) [*gst-2, gst-3, gst-5, gst-11, gst-21, gst-29, gst-30*], and *nlg-1* (tm474) [*gst-12*]. Upregulation in glutathione peroxidases genes were also observed in the *nrx-1* (ok1649) [*gpx-1, gpx-6*], *nrx-1* (tm1961) [*gpx-6, gpx-8*], and the *nlg-1* (ok259) [*gpx-6*] strains. Additionally, the *nrx-1* (tm1961) strain showed upregulation of dehydrogenase, short-chain genes (*dhs-1, dhs-7, dhs-11, dhs-18, dhs-19, dhs-20, dhs-21, dhs-22*), which enable oxidoreductase activity and regulate reactive oxygen species. Conversely, downregulation of heat shock protein genes (*hsp-1, hsp-3, hsp-6, hsp-12.2, hsp-60*) was observed. These genes enable heat shock protein binding activity and are involved in protein refolding and response to heat. Furthermore, *nrx-1* (tm1961) mutation also resulted in downregulation of chaperonin-containing T-complex polypeptide (TCP) genes (*cct-1*, *cct-2*, *cct-5*), which enable unfolded protein binding activity, and are involved in protein folding. Other genes invoved in immune responses were also upregulated across all strains, as follows: *nrx-1* (ok1649) [*cnc-2*, *nhr-115*, *prx-11*, C29F3.7, C32H11.4, C55A6.7, *ugt-44*, F01D5.3, *irg-4*, *dct-17*, F35E12.9, F35E12.10, F44G3.10, F54D5.4, F55G11.8, T24B8.5, ZK228.4, C17H12.6, *maoc-1*, H20E11.1, H20E11.2, H20E11.3], *nrx-1* (tm1961) [*aqp-10*, *prx-11*, *sod-2*, *try-3*, C08E8.4, C55A6.7, E04D5.4, *ugt-44*, F16H6.10, F44G3.10, *dod-22*, *fbxa-30*, *fbxa-105*, *irg-1*, *maoc-1*, H20E11.1, H20E11.2], *nlg-1* (ok259) [C10C5.2, C32H11.3, ugt-44, F44G3.10, dod-22, F55G11.8, ZK218.7, *irg-1*, C17H12.6, C17H12.8, *fbxa-60*], and *nlg-1* (tm474) [C32H11.4, F01D5.3, *dct-17*].

### *Nrx-1/nlg-1* mutations downregulate genes involved in neurotransmission and neuronal signaling

Downregulation of FMRF-like peptide genes, which enable neuropeptide receptor binding activity and are involved in the neuropeptide signaling pathway, was notably observed in *nrx-1* (ok1649) [*flp-13, flp-15*], *nlg-1* (ok259) [*flp-3, flp-11, flp-13, flp-15, flp-21*], and *nlg-1* (tm474) [*flp-10*] strains. *Nrx-1* (tm1961) strain showed downregulation of uncoordinated genes (*unc-11, unc-15, unc-17, unc-29, unc-34, unc-50, unc-52, unc-57, unc-60, unc-62, unc-69*), which enable signaling receptor binding activity and are involved in neuromuscular synaptic transmission, axon development, and nematode development. The *nlg-1* (ok259) mutation resulted in downregulation of neuropeptide-like protein genes (*nlp-6, nlp-9, nlp-21*).

### *Nrx-1/nlg-1* loss alters genes involved in transcriptional regulation

DEGs analysis of mutant strains showed downregulation of histone genes in *nrx-1* (ok1649) [*his-11, his16, his-24, his-71*] and *nlg-1* (ok259) [*his-71*] strains, which are predicted to be structural components of chromatin. *Nrx-1* (tm1961) mutation resulted in downregulation of large and small subunits of ribosomal protein genes (*rpl-1, rpl-2, rpl-3, rpl-4, rpl-5, rpl-6, rpl-7, rpl-7A, rpl-9, rpl-12, rpl-13. rpl-15, rpl-16, rpl-17, rpl-18, rpl-19, rpl-20, rpl-24.1, rpl-25.1, rpl-25.2, rpl-26, rpl-28, rpl-30, rpl-31, rpl-33, rpl-34, rpl-35, rps-0, rps-1, rps-3, rps-4, rps-5, rps-6, rps-7, rps-8, rps-10, rps-11, rps-12, rps-13, rps-14, rps-16, rps-18, rps-19, rps-21, rps-22, rps-26, rps-27, rps-29, rps-30, rsp-2, rsp-7, rsp-8*), as well as *nlg-1* (ok259) mutation (*rpl-14*, *rpl-23*, *rpl-25.1*, *rpl-32*, *rps-21*, *rps-25*). These genes enable RNA-binding activity and are predicted to be a structural constituent of ribosomes. *Nrx-1* (tm1961) mutation resulted in downregulation of abnormal cell lineage genes (*lin-10*, *lin-11*, *lin-13*, *lin-36*, *lin-40*, *lin-41*, *lin-53*), which enable DNA-binding transcription factor activity, and regulate neurogenesis and neuron-neuron synaptic transmission. It also resulted in upregulation of nuclear hormone receptor family of genes (*nhr-11*, *nhr-12*, *nhr-42*, *nhr-44*, *nhr-69*, *nhr-92*, *nhr-100*, *nhr-101*, *nhr-102*, *nhr-104*, *nhr-107*, *nhr-109*, *nhr-115*, *nhr-136*), which are predicted to enable DNA-binding transcription factor activity (Supplementary file 2).

### *Nrx-1/nlg-1* mutation alters genes involved in mitochondrial organization and homeostasis

*Nrx-1* (ok1649) mutation resulted in the upregulation of genes involved in mitochondrial organization, including *coq-5*, *stl-1*, and the downregulation of adenine nucleotide translocator genes (*ant-1.3*, *ant-1.4*), which are involved in mitochondrial ATP transmembrane transport, and regulation of mitochondrial membrane permeability. Furthermore, upregulation of small and large subunits of mitochondrial ribosomal protein genes (*mrps-15*, *mrpl-9*, *mrpl-2*3) was also observed. Similarly, *nrx-1* (tm1961) mutation resulted in upregulation of small and large subunits of mitochondrial ribosomal protein genes (*mrpl-9*, *mrpl-13*, *mrpl-18*, *mrpl-23*, *mrpl-32*, *mrpl-40*, *mrpl-41*, *mrpl-46*, *mrpl-50*, *mrpl-51*, *mrpl-54*, *mrps-2*, *mrps-13*, *mrps-17*, *mrps-18A*, *mrps-18B*, *mrps-24*), and other family of genes involved in mitochondrial electron transport (*ctc-3*, *ctb-1*, *nduo-1*, *nduo-2*, *nduo-3*, *nduo-4*, *nduo-5, nduo-6*, *ndfl-4*). Downregulation of adenine nucleotide translocator genes (*ant-1.1*, *ant-1.3*, *ant-1.4*) involved in mitochondrial organization was also observed. *Nlg-1* (ok259) mutation resulted in the upregulation of genes involved in mitochondrial organization, including *coq-5*, *stl-1*, as well as the downregulation of adenine nucleotide translocator genes (*ant-1.3*, *ant-1.4*), which are involved in mitochondrial ATP transmembrane transport and regulation of mitochondrial membrane permeability. Furthermore, this mutation also resulted in upregulation of small and large subunits of mitochondrial ribosomal genes (*mrpl-9*, *mrpl-15*, *mrpl-18*, *mrpl-24*, *mrpl-50*, *mrps-2*, *mrps-12*, *mrps-15*).

### Identification of co-regulated gene clusters following nrx-1/nlg-1 mutation

**Figure 3:**
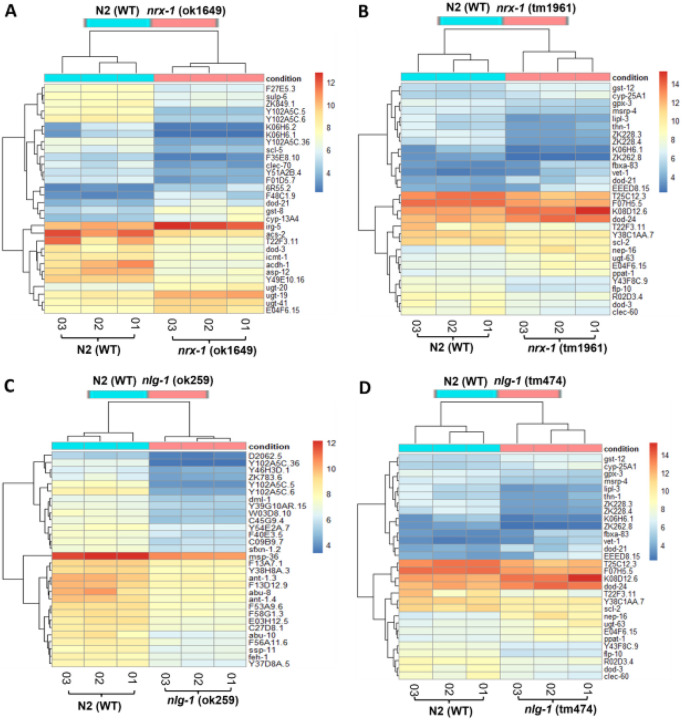
Bi-clustering of the top 30 DEGs for all mutant strains relative to WT. Degree of statistical significance is indicated with color patterns, with red indicating high statistical significance, while blue indicates lower significance closer to 0.05.

Bi-clustering heatmaps were used to identify co-regulated genes for each strain. Their expression profile was sorted by adjusted p-value (Fig. 3). This was done by plotting their log2-transformed expression values. The variations in transcriptomics profiles observed across the four mutant strains, particularly the *Nrx-1* (tm1961) strain, suggest gene-related activation of specific pathways. Principal component analysis (PCA) was used to reveal similarities between worm samples based on the distance matrix (Fig. 2A–D). The samples were projected to a 2D plane spanned by their first two principal components, revealing the overall experimental covariates and batch effects. For all mutant strain comparisons to the wild-type control, the x-axis direction explained the most variance, while the y-axis explained the second most. PCA for *nrx-*1 (ok1649) showed 88% of the total variance (PC1 51%, PC2 37%), *nrx*-1 (tm1961) showed 93% (PC1 84%, PC2 9%), *nlg*-1 (ok259) showed 94% (PC1 69% PC2 25%), and *nlg*-1 (tm474) showed 92% (PC1 88% PC2 11%) (Fig. 2A–D).

### Gene Ontology reveals nrx-1/nlg-1 mutation impacts on C. elegans development, metabolism, and signaling pathways

**Figure 4:**
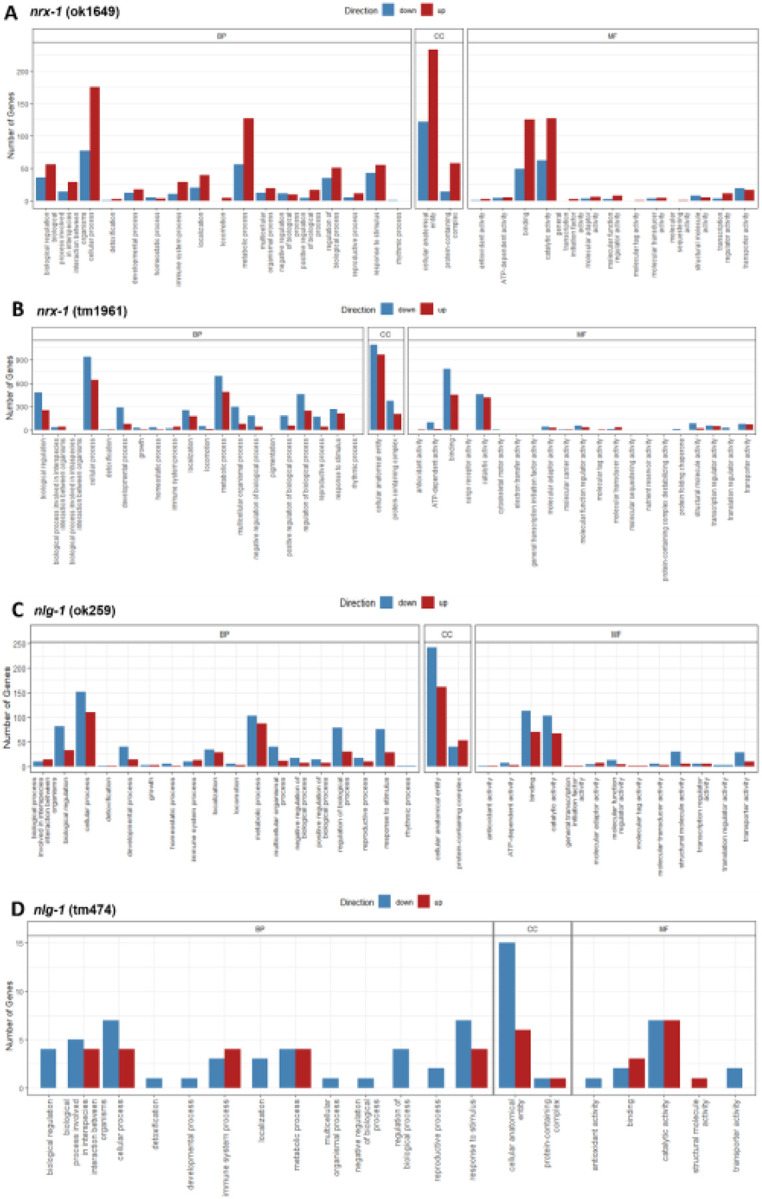
GO Functional Analysis of DEGs in *nrx-1* and *nlg-1* Mutants. Functional analysis of Biological Processes (BP), Cellular Components (CC), and Molecular Functions (MF) for the following strains: (A) *nrx-1* (ok1649), (B) *nrx-1* (tm1961), (C) *nlg-1* (ok259), and (D) *nlg-1*(tm474).

GO analysis revealed significant biological processes across all worm strains (Supplementary file 2), including pathways involved in development, metabolism, and signaling (Fig. 4). Significant DEGs were clustered by their gene ontology, and the enrichment of gene ontology terms was tested using Fisher's exact test (GeneSCF v1.1-p2). Figure 4 show gene ontology terms that are significantly enriched. Enrichment analysis of DEGs was also conducted, and it revealed significant effects of *nrx-1/nlg-1* mutations on key biological processes in the *C. elegans*, particularly in biological process (BP), molecular function (MF), and cellular component (CC). Worms with the LOF mutation in the *nrx*-1 (ok1649) were enriched in innate immune response (Fig. 4A), while the *nrx-1* (tm1961) strain was enriched in translation and negative regulation of translation (Fig. 4B).

The *nlg*-1 (ok259) strain was enriched in endoplasmic reticulum UPR and peptidyl-threonine phosphorylation (Fig. 4C). The *nlg-*1 (tm474) strain was enriched in innate immune response, defense response to gram-negative bacterium, negative regulation of oviposition, defense response to gram-positive bacterium, de novo GDP-I-fructose biosynthetic process, GDP-I-fructose biosynthetic process, GDP-mannose metabolic process, peptidoglycan catabolic process, purine nucleobase biosynthetic process, de novo IMP biosynthetic process, NADH oxidation, and others (Fig. 4D).

### KEGG enrichment and PEA reveal impact of nrx-1/nlg-1 mutation on C. elegans metabolism, signaling, and aging pathways

**Figure 5:**
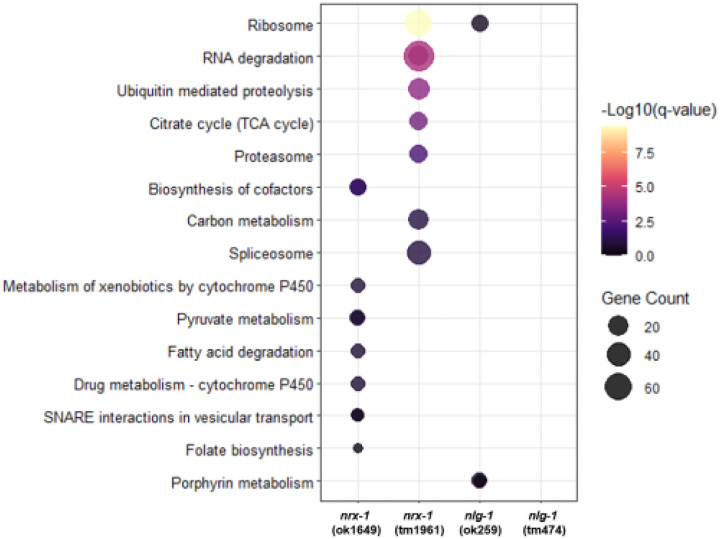
Top significantly enriched KEGG pathways of DEGs following *nrx-1/nlg-1* mutations in *C. elegans*. The four mutants are compared on the abscissa, while the enrichment pathways are displayed on the ordinate. Gene counts are represented by the size of the dot; the larger the dot, the larger the number of genes enriched in that particular pathway. The color of the dot represents the significance value of the enrichment of the pathways. Yellow indicates more significance.

All DEGs across strains were compared and analyzed for their Kyoto Encyclopedia of Genes and Genomes (KEGG) pathway using the Metascape database^38^. The enrichment results were sorted by *p*-value from smallest to largest, and the top 15 items are presented in Fig. 5. KEGG analysis showed the impacts of the *nrx-1 or nlg-1* mutation on key metabolism and signaling pathways in *C. elegans*. Following the *nrx-1* (*ok1649*) mutation, the following KEGG pathways were enriched by DEGs: biosynthesis of cofactors, metabolism of xenobiotics by cytochrome P450, pyruvate metabolism, fatty acid degradation, SNARE interactions in vesicular transport, and folate biosynthesis. *Nrx-1* (tm1961) mutation enriched the following KEGG pathways: ribosome, RNA degradation, ubiquitin-mediated proteolysis, citrate cycle (TCA cycle), proteasome, carbon metabolism, and spliceosome. *Nlg-1* (ok259) mutation enriched the ribosome and porphyrin metabolism pathways. Lastly, no KEGG pathway was enriched following the *nlg-1* (tm747) mutation in the worms (Fig. 5). Protein enrichment analysis (PEA) was employed to reveal observable phenotypic traits and disease conditions associated with significant DEGs. PEA for *nlg-1* (tm474) failed to produce significant observable phenotypes based on DEGs. PEAs for *nrx*-1 (tm1961), *nrx*-1 (ok1649), and *nlg*-1 (ok259), when compared to wild-type control, are presented in Supplementary Fig. 4 (S4).

## Discussion

It is now being established that synaptic genes modulate neurodevelopmental processes in *C. elegans*. Neurexin and neuroligin proteins are key synaptic partners that facilitate communication between neurons while maintaining neuronal integrity. This study aimed to delineate genome-wide transcriptomic changes associated with loss-of-function mutations in *nrx-1* and *nlg-1* (homologs of human *NRXN* and *NLGN*, respectively) in *Caenorhabditis elegans*, and to relate these changes to observable phenotypic outcomes. This integrative approach provides a framework for understanding how neurexin and neuroligin loss of function influences development and behavior.

Transcriptomics analysis identified DEGs and key signaling and metabolic pathways that are associated with the *nrx-1/nlg-1* mutation in *C. elegans*. Notably, the *nrx-1* (tm1961) mutation showed a more widespread transcriptomic alteration when compared to the other mutant strains.

Post-embryonic development in *C. elegans* is marked by four molting periods, which are timed processes involving the shedding of a collagen-rich outer cuticle^39^. This ensures the transition from one larval stage to the next, hence facilitating growth in the worms^40^. We asked an important question regarding how collagen expression impacts the growth of the worms. Collagen genes, which are actively involved in collagen and cuticle development, were consistently downregulated in the *nrx-1* (ok1649), *nrx-1* (tm1961), and *nlg-1* (ok259) strains. This points to potential impairment of collagen and cuticle synthesis, as well as disruption of molting, which could account for the shorter body length of worms observed in this study. A recent study suggested a functional connection between the expression of collagen genes and longevity, as downregulation of these genes affected the lifespans of the worms, particularly under stressful conditions. Their study attributed the reduction in cuticle collagen transcripts to an age-associated decline in adult extracellular matrix maintenance/repair programs^41^. The *dpy-4* and *dpy-13* genes were also downregulated, particularly in the *nlg-1* (ok259) and *nrx-1* (tm1961) strains. These genes are also integral to maintaining the cuticle-based molting cycle, suggesting that their downregulation could play a part in the observed molting deficits and the resultant shorter body length in these strains. DPY mutant worms exhibit a ‘dumpy phenotype’, characterized by abnormally short and fat bodies^42^. This finding was further buttressed by Goodman and Savage-Dunn^43^ who showed that defects in a subset of *C. elegans* collagen genes resulted in a visible dumpy phenotype in which mutants are shorter and wider than wild-type animals. In their study, mutations in the first class of dpy genes (*dpy-2*, *dpy3*, *dpy-7*, *dpy-8*, or *dpy-10*) produced furrowless mutants. They suggested that genes encoding cuticle collagens determine body size and shape, not only the fine structure of the cuticle. Actin genes, which are essential for cytoskeletal organization, were also downregulated in the *nrx-1* (tm1961) strain. These genes are critical for cuticle synthesis and contribute to the integrity of the worm’s body circumference^44^, and their disruption has been shown to result to developmental deficits, including the failure of *C. elegans* embryos to elongate during development, resulting to shorter worms^45^. Furthermore, the lethal group of genes that has been shown to regulate larval-adult transition and other developmental timing processes^46^ was also downregulated in the *nrx-1* (tm1961) strain. Taken together, these dysregulations of critical genes may underlie the observed developmental deficits following these mutations. Given these findings, recent therapeutic interventions have focused on maintaining collagen in biological systems, as their disruption compromises development and lifespan^47,48^.

**Figure 6:**
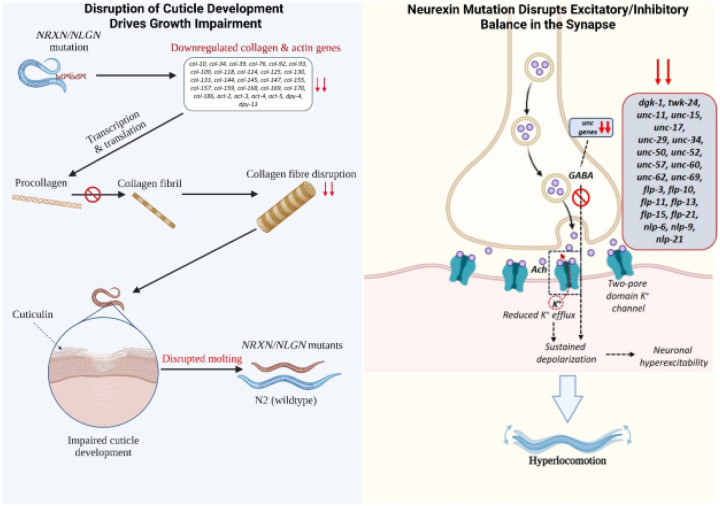
Schema of system-wide transcriptional reprogramming following loss of neurexin and neuroligin. Loss of *nrx-1* and *nlg-1* disrupts synaptic function and triggers coordinated transcriptional changes across multiple biological systems. Downregulation of genes involved in collagen and cuticle development impairs molting and organismal growth, while reduced expression of synaptic and neuronal signaling genes disrupts excitatory/inhibitory balance, contributing to altered locomotion and behavior. Concurrent activation of stress-response, immune, and metabolic pathways reflects a shift toward a stress-adaptive physiological state. Together, these changes are consistent with the idea that synaptic gene disruption is associated with molecular alterations and organism-level phenotypes (created in BioRender).

Coordinated movement in worms is brought about by consistent patterns of alternating dorsal and ventral contractions, which are regulated by interactions between excitatory and inhibitory neurons. Excitatory neurons release acetylcholine, which contracts the body wall muscle on one side and stimulates the GABAergic neurons to release GABA on the opposing muscle wall^49^. Disruption to this process could impair coordinated muscle contraction and movement in the worms. Here, we observed that the two mutant strains showed contrasting locomotion behavioral phenotypes when compared to the control. This points to defective neuromuscular signaling in the mutant strains, particularly the *nrx-1*-deficient mutants, which consistently showed hyperlocomotion and hyper-repetitive phenotypes. This suggests excitatory/inhibitory imbalance and deficits in synaptic transmission. The *Nrx-1* (tm1961) strain showed downregulation of the *unc* family of uncoordinated genes, which are involved in neuromuscular synaptic transmission^50^. These *unc* genes are required for synthesizing GABA^51,52^, and their downregulation could mean more excitatory neuronal activity^53^. This could contribute to the observed hyperlocomotion observed in this study (Fig. 6). Furthermore, the *dgk-1* and the *twk-24* genes were also downregulated. The *twk-24*, like other members of the *twk* family, governs locomotion and neuronal excitability, and a loss of function of these genes has been shown to result in a hyperactive state^54,55^. Gottschling, et al.^54^’s study showed that loss of the *twk-7* gene caused hyperactivity resulting from hyperexcitation of muscle membranes. In their study, the loss-of-function mutants of *dgk-1* also exhibited hyperactivity on agar plates. Consistent with our findings, Calahorro and Ruiz-Rubio^56^ showed a similar hyper-repetitive phenotype in neurexin mutants and hypo-repetition in the neuroligin mutants. Supplementation with human alpha- or beta-NRXN1 isoforms in the neurexin-deficient mutants rescued the locomotion deficits in the worms. In another study, *nrx-1* deletion was shown to impair cholinergic neurotransmission onto GABAergic neurons^57^. Bhat, et al.^58^ showed that mutants in the neuropeptide *flp-15* had large increases in the mean amplitude of body bends, indicating defects in the locomotory state of these animals. They further suggested that FLP-15 functions through NPR-3 to regulate the amplitude of body bends. On the other hand, the downregulation of the *unc* genes could contribute to the hypo-locomotion observed in the neuroligin strains, as these genes are important for priming synaptic vesicles for release. The *unc-13* is a typical example, and its downregulation has been previously shown to disrupt the release of neurotransmitters at neuromuscular junctions^59^. When neurotransmitter release is impaired, movement is also negatively affected. Furthermore, these genes contribute to electrical coupling in the synapse; for example, the *unc-1* gene regulates gap junctions that are formed by *unc-9* between muscles and neurons. Loss of both the *unc-1* and *unc-9* genes reduces electrical coupling and causes uncoordinated movement in the worms, contributing to the hypolocomotion phenotype seen in the neuroligin strains^60^.

Dysregulation in immune response was one of the most prominent changes observed from the transcriptomics analysis. Several genes involved in the innate immune response were significantly upregulated in *nrx-1/nlg-1* mutants. Both mutations, however, showed dysregulation in glutathione S-transferase (GST) and glutathione peroxidase genes when compared to the control. These genes are part of the immune system response that functions to maintain cellular homeostasis, oxidoreductase activity, and regulate reactive oxygen species. Although this response is intended to restore immune and homeostatic balance, usually via detoxification, it can become maladaptive and consequently drive inflammatory responses that lead to autoimmune and autoinflammatory diseases^61^. Recently, studies have begun to explore the mechanisms underlying GST overexpression in tumor drug resistance and neurodegenerative diseases. While some studies have revealed that the overexpression of some GSTs inhibits neurodegeneration^62,63^, others have shown that GSTO1 could mediate inflammatory response and contribute to the pathogenesis of Parkinson’s and Alzheimer’s disease^64,65^. High GSTP1 expression has been found in the neuroglia of epileptic foci in brain specimens from patients with refractory epilepsy, when compared to patients with non-refractory epilepsy^66^.

Wild-type worms feed in isolation in the presence of abundant bacteria. While all four mutant strains exhibited abnormal increases in clumping and bordering behavior, the greatest effect was observed in the *nrx-1* (tm1961) strain. We suggest this resulted from the downregulation of FMRF-like peptide genes, which enable neuropeptide receptor binding activity and are involved in the neuropeptide signaling pathway. They also encode ligands that activate the NPR-1 receptor, and low levels have been shown to trigger clumping^67^. We also observed a downregulation of neuropeptide-like protein genes in the *nlg-1* (ok259) strain. These neuropeptides are major signaling molecules that influence neural circuits and, consequently, enhance clumping behavior^68^. Earlier investigations by de Bono and Bargmann^69^ showed how the neuropeptide receptor 1 mutation results in abnormal clumping and bordering behavior in the worms^69,70^. Neurexin mutations have also been shown to act on ASH and ADL sensory neurons, which increases the clumping rate^71^. This aggregate feeding behavior is regulated by conserved autism-associated genes, including *nrx-1*, *nlg-1*, *glr-1glr-2*^71^.

Studying VNC development in *C. elegans* provides insights into how axons develop and find their path. Some neurons exhibit specific gene expression profiles that reveal a differentiated state at a particular developmental stage^72^. In the synaptic wiring of VNC neurons of *C. elegans*, gene expression is mediated through hormones and their effector transcription factors to result in the structural remodeling of individual groups of neurons^73^. We identified some genes that could impact molecular signaling pathways and affect how these VNC neurons are structured. The *lim-6*, *lim-7*^74^, and *ceh-14*^75^ have been identified as key players in VNC development during the embryonic stage, while the *zig* family of genes is crucial for post-embryonic VNC development^76^. The absence or reduced expression of the *zig* family of genes has been shown to cause defects in the positioning and maintenance of axons in the VNC^76^. Notably, we found that *lim-8, lim-7*, *ceh-14*, *ceh-34*, *ceh-37*, and *ceh-100* genes, which are essential for the development of the VNC, were significantly downregulated in the *nrx-1* (tm1961) strain. We suggest that this delayed VNC developmental timing may contribute to the impaired development of the VNC, although the post-embryonic genes (*zig-10*, *zig-11*, and *zig-6*) were not downregulated. The other strains showed a similar reduction in VNC viability and size. However, we suggest that similar mechanisms are responsible for this, albeit not as significantly as observed in the *nrx-1* (tm1961) strain. Furthermore, downregulation of large and small subunits of ribosomal protein (RP) genes, cell lineage genes, and nuclear hormone receptor genes was also observed in this study, particularly in the *nrx-1* (tm1961) strain. These genes are also essential for VNC development and maturation. RP genes trigger translational mechanisms needed for rapid cell division during VNC formation^77^. Their decreased expression may contribute to the reduced VNC size observed in the present study.

The *C. elegans* relies on mitochondria for ATP production, lipid metabolism, and apoptosis^78^. Although mitochondria can be found in almost all somatic cells, they are denser in energy-demanding tissues of the worm’s body, particularly the muscle tissues, germline, and intestine. Among the various functions mitochondria perform in the worms, they are particularly important for regulating aging, lifespan, stress response, and apoptosis^78^. In this study, both neurexin and neuroligin mutants consistently showed upregulation in genes involved in mitochondrial organization, including *coq-5*, *stl-1*, and several mitochondrial ribosomal protein genes (*mrps* and *mrpl* family members). This points to a potential compensatory response, where the over-expression of these genes may be associated with altered mitochondrial function. This compensation could be in the form of increased production of proteins necessary for the electron transport chain's efficiency. Conversely, we also observed consistent downregulation of adenine nucleotide translocator (ANT) genes in both strains, which suggests a potential deficit in the energy delivery system. ANT moves ADP into the mitochondria and ATP out to all cells and tissues. This suggests an energy transfer deficit due to downregulation of ANT genes, which may impact various systems, including the muscles, gut, and neuronal systems.

GO enrichment analysis showed that the *nrx-1/nlg-1* mutation greatly influenced a range of biological processes and molecular functions, including biological regulation, cellular and developmental processes, immune and metabolic pathways, response to stimulus, localization, and reproductive processes, which include fertilization, gamete formation, and zygote development. At the molecular function level, significant impacts were observed in binding, catalytic activity, and transporter activity.

### Conclusion:

In summary, this study revealed novel molecular and behavioral alterations resulting from LOF mutations of *nrx-1/nlg-1* in *C. elegans*. Here, we reveal a more pronounced impact of the neurexin mutation on the worms, with this mutation resulting in the most significant DEGs and disruptions in their associated functions, including cuticle development, synaptic transmission, mitochondrial function, DNA/RNA binding, and neuronal growth. We also reveal key genes that could be associated with the observed phenotypic changes in development, locomotion, repetition, sociality, and neuronal morphology.

### Limitations:

We acknowledge a potential background modifier effect as the *nrx-1* (tm1961), *nlg-1* (ok259), and *nlg-1* (tm474) mutant alleles, which were sourced from different genetic backgrounds, were not backcrossed before transcriptomic sequencing. This study is also limited by its reliance on transcriptomic analyses to infer functional changes, and future work will be required to establish causal links between gene expression and phenotype. Further, while *C. elegans* provides a powerful and highly conserved model for studying fundamental biological processes; extending these findings to more complex systems will further refine their relevance to neurodevelopmental disorders.

## Methods

### C. elegans Strains and Maintenance

The following strains were used: N2 wildtype (*wt*), *nrx-1 (ok1649)*, and *nlg-1 (ok259)* strains (obtained from the Caenorhabditis Genetics Center (CGC), USA), *nlg-1(tm474) and nrx-1 (tm1961)* strains (sourced from the National Bioresource Project (NBRP), Japan), allowing the study of different mutant alleles for each gene. The *nrx-1* (ok1649) strain mutation consists of an 861 bp deletion, which impacts the long alpha isoforms. The tm1961 allele consists of a 428 bp deletion that targets the long alpha isoform transcripts of nrx-1 only. The *nlg-1* (ok259) had a full knockout, while the *nlg-1* (tm474) strain had a 583 bp deletion within the C40C9.5 locus.

All *C. elegans* strains were handled and maintained at 20°C on Nematode Growth Medium (NGM) agar plates seeded with *Escherichia coli* OP50 as a food source. Worms were transferred periodically onto fresh NGM plates using a sterile worm pick or agar chunking technique to ensure healthy propagation. For all experiments, worms were initially grown on NGM containing 8-fold peptone for 2–3 days. Then, aged-synchronized L1 populations were obtained using the standard hypochlorite protocol as previously described, washed in 85 mM NaCl buffer solution, and transferred to standard NGM plates seeded with *E. coli* OP50^79^. Worms were allowed to grow for 48 hours at 20°C to the young adult stage (48 hours post-L1) and were collected for all experiments described below.

### Assessment of growth rate, behaviors, and neuromorphology

#### Measurement of Body Length

This assay of body length was measured to assess growth rate. Here, worms grown to young adults were washed with M9 buffer and collected onto a microscope slide on 2% agarose pad, paralyzed with 3 mM levamisole (L9756; Sigma-Aldrich, USA). Images are obtained using a Nikon SMZ745T Trinocular stereomicroscope (Nikon Instruments, USA) equipped with a View4K high-definition camera and are imported into ImageJ for scoring, for measurement of body length by drawing a freehand line from the head to the tip of the tail along the worm midline axis. The assay was repeated in four independent worm preparations (n = 25–30 worms per replicate). All behavioral assays were later scored by trained observers in a blinded manner.

##### Locomotor Assay:

Locomotion was assessed, as we have established previously^79^. Synchronized L1 worms were grown on NGM plates and collected at the young adult stage. Worms were washed with S-basal buffer (composition: 5.85 g NaCl, 1 g K_2_HPO_4_, 6 g KH_2_PO_4_, 1 mL cholesterol [5 mg/mL in ethanol] per 1 L). Worms were transferred onto unseeded assay plates in drops of S-basal. Excess liquid was removed with Kimwipes, and the worms were allowed to acclimate for 5 minutes. Locomotor activity was quantified by counting body bends for 3 minutes. Data are expressed as body bends per 3 minutes. The assay was repeated in four independent worm preparations (n = 25–30 worms per replicate).

#### Repetitive Behavior Assay

The assay was carried out using the already established protocols of Balasingam and Messenger^80^. Repetitive behavior was defined as a head movement followed by a tail movement and another head movement (“head-tail-head” sequence), resulting in repetitive, stationary movements. Only spontaneous behaviors were counted; movements altered by contact with other worms were excluded to minimize variability. Worms were washed off a seeded plate into a siliconized 1.5 mL microcentrifuge tube and rinsed 2–3 times with M9 buffer to remove bacterial debris. They were then transferred onto fresh, unseeded NGM plates. The movements were observed for 1 minute, and the frequency of “head-tail-head” sequences was recorded per strain. The assay was repeated in four independent worm preparations (n = 25–30 worms per replicate).

#### Clumping and Bordering Behavior

Clumping and bordering behavior assays were done as stated by^69,81^. Six-centimeter NGM plates (2.1% agar) were seeded 24 hours prior with 50 μL OP50 in LB medium, producing a 10-mm diameter lawn. Between ~ 300–700 well-fed young adult worms were placed outside the lawn and allowed to roam for 3 hours at 20°C. After incubation, the percentage of worms exhibiting clumping (aggregation) and bordering (remaining at the lawn edge) was quantified from captured images. Images are obtained using a Nikon SMZ745T Trinocular stereomicroscope (Nikon Instruments, USA) equipped with a View4K high-definition camera and are imported into ImageJ for scoring. To ensure consistent quantification across all strains, a standardized grid overlay was applied to all images using ImageJ. This allowed for consistent quantification of worm distribution across all experimental plates. The total number of worms, clumped worms, and bordered worms was recorded using an ImageJ Cell Counter plugin. Here, we used the *npr-1(ad609) X* strain with loss of *npr-1*, causing worms to aggregate (clump and borders), as a positive control. The percentage (%) of worms in clumps or borders was calculated and used for data analysis. Experiments were repeated with 4 independent worm preparations.

#### DAPI Staining for Nuclear Morphology

DAPI (4′,6-diamidino-2-phenylindole) staining was used to assess nuclear integrity and count nuclei, following previously established protocols^82^. Briefly, young adult worms grown from synchronous L1 plates were collected and rinsed with M9 buffer. The worms were collected as pellets through centrifugation at 1000 × g for 60 seconds, and the supernatant was carefully removed. Worms were washed with 1 mL of distilled water, then fixed in 400 μL of 30% acetone for 15 minutes. After fixation, samples were centrifuged and washed twice with 500 μL distilled water. Worms were stained with 200 μL DAPI solution (10 μg/mL) for 15 minutes, followed by two washes. Stained worms were mounted on glass slides with 2% agar pad and covered with coverslips. Thereafter, worms were imaged under the Leica DM750 Fluorescence Microscope, imported to ImageJ for digital segmentation of neurons of the ventral nerve cord (VNC). The VNC neurons were distinguished from other non-neuronal cells by their typical smaller and highly condense appearance. Also, they appear in a ladder-like chain of repeating neuron pool on the ventral hypodermal ridge. We scored the number of viable VNC neurons by assessing nuclear morphology and size according to established methods^83,84^. Thereafter, 10–15 VNC neurons per worm were measured with the oval selection tool in ImageJ, and their averages were recorded. The assay was repeated in four independent worm preparations (n = 25–30 worms per replicate).

### Statistical analysis of body length, behavior, and neuromorphology

Analysis was performed using GraphPad Prism 8.0.2 (GraphPad Software, San Diego, CA, USA). Differences between groups were determined via one-way ANOVA, followed by Dunnett’s post hoc test for multiple comparisons. A *P* < 0.05 was considered statistically significant.

### Sample preparation for RNA-Seq

For -omics analysis, aged-synchronous nematodes were collected at the young adult stage from NGM plates. Approximately 20,000 worms were collected into 15 mL tubes with a solution of 85 mM NaCl with 0.1% Tween 20 to facilitate the removal of bacterial films, followed by a single wash in this solution. Subsequently, the samples were washed three times with 85 mM NaCl alone to remove residual debris. The worms were then transferred to 2.0 mL tubes, snap-frozen in liquid nitrogen, and stored at − 80°C. Samples were collected from three independent worm preparation/replicates. Samples were shipped on dry ice to GENEWIZ-Azenta Life Sciences (NJ, USA) for RNA-Sequencing.

### Total RNA-Sequencing Experiment

Information in this section is provided by GENEWIZ-Azenta Life Sciences, unless otherwise stated, when authors have performed additional analysis.

#### RNA extraction

Total RNA was extracted from the resulting lysates using the RNeasy Plus Universal Mini Kit (Qiagen) according to the manufacturer’s instructions. RNA concentration was quantified using a Qubit 4.0 Fluorometer (Life Technologies, Carlsbad, CA, USA), and RNA integrity was assessed using an Agilent 4200 TapeStation system (Agilent Technologies, Palo Alto, CA, USA).

#### Library Preparation and Sequencing

RNA sequencing libraries were prepared and sequenced by GENEWIZ-Azenta (South Plainfield, NJ, USA). Briefly, rRNA depletion was performed using the QIAGEN FastSelect rRNA HMR Kit (Qiagen, Germantown, MD, USA). Strand-specific libraries were constructed using the NEBNext Ultra II Directional RNA Library Prep Kit (NEB, Ipswich, MA, USA) following the manufacturer’s instructions. The enriched RNAs were fragmented for 8 minutes at 94°C. First-strand and second-strand cDNA were subsequently synthesized. The second strand of cDNA was marked by incorporating dUTP during the synthesis. cDNA fragments were adenylated at 3’ends, and an indexed adapter was ligated to the cDNA fragments. Limited-cycle PCR was used for library enrichment. The incorporated dUTP in the second-strand cDNA quenched the amplification of the second strand, which helped to preserve the strand specificity. The sequencing library was validated on the Agilent TapeStation (Agilent Technologies, Palo Alto, CA, USA) and quantified by using Qubit 4.0 Fluorometer (ThermoFisher Scientific, Waltham, MA, USA) as well as by quantitative PCR (KAPA Biosystems, Wilmington, MA, USA). The libraries were clustered on a flowcell and sequenced on an Illumina NovaSeq X Plus platform in a 2×150 bp paired-end configuration. Raw sequence data were converted to fastq files and de-multiplexed using Illumina's bcl2fastq 2.20 software.

#### Transcriptomics Data Analysis

Raw sequence reads were processed to remove low-quality bases and adapter sequences using Trimmomatic (v.0.36). High-quality, trimmed reads were then aligned to the *Caenorhabditis elegans* reference genome (available on ENSEMBL) using the STAR aligner (v.2.5.2b). To quantify gene expression, we used the feature Counts function within the Subread package (v.1.5.2), focusing on uniquely mapped reads that aligned to annotated exon regions. The resulting raw gene hit counts were used for differential expression analysis using the DESeq2 R package.

#### Statistical Analysis and Visualization

Comparisons between experimental groups were performed using the Wald test to calculate P-values, which were subsequently adjusted for multiple testing. Genes were classified as differentially expressed (DEGs) if they met the criteria of an adjusted P-value < 0.05 and an absolute log2 fold change > 1. Principal Component Analysis (PCA) was visualized using the plotPCA function in DESeq2, with the plot generated using the top 500 genes ranked by row variance. The data analyzed from GENEWIZ-Azenta was further processed for Gene Ontology (GO) analysis and visualizations on R version 4.3.1 (R Foundation) by the authors. GO functional classification analysis was performed using the clusterProfiler and org.Ce.eg.db R packages to categorize DEGs into Biological Process (BP), Molecular Function (MF), and Cellular Component (CC) ontologies. Further, the authors performed Kyoto Encyclopedia of Genes and Genomes (KEGG) pathway analysis using the Metascape database^38^. The enrichment results were sorted by *p*-value (P < 0.05) from smallest to largest, and the top 15 items are presented.

## Supplementary Material

This is a list of supplementary files associated with this preprint. Click to download.

- Ijomoneetal2026NatureStyleSupplInfo.docx
- Ijomoneetal2026SeqSupplementaryFile.xlsx

## Data Availability

The data supporting the findings of this study are available from the corresponding authors upon request. Sequencing data have been deposited in GEO (Gene Expression Omnibus) under accession number GSE330481. Data for the figures and supplementary figures are provided as a supplementary file.
